# Inequality in physical activity, global trends by income inequality and gender in adults

**DOI:** 10.1186/s12966-020-01039-x

**Published:** 2020-11-26

**Authors:** Chastin SFM, J. Van Cauwenberg, L. Maenhout, G. Cardon, E. V. Lambert, D. Van Dyck

**Affiliations:** 1 School of Health and Life Sciences, Glasgow Caledonian University, Glasgow, UK; 2 Department of Movement and Sports Sciences, Ghent University, Ghent, Belgium; 3 Department of Public Health and Primary Care, Ghent University, Ghent, Belgium; 4 Health through Physical Activity, Lifestyle and Sport Research Centre (HPALS), Department of Human Biology, Faculty of Health Sciences, University of Cape Town, Cape Town, South Africa

**Keywords:** Economy, Inactivity, Guidelines, Physical activity, Gini index, Inequality, WHO activity guidelines

## Abstract

**Background:**

Physical inactivity is a global pandemic associated with a high burden of disease and premature mortality. There is also a trend in growing economic inequalities which impacts population health. There is no global analysis of the relationship between income inequality and population levels of physical inactivity.

**Methods:**

Two thousand sixteen World Health Organisation’s country level data about compliance with the 2010 global physical activity guidelines were analysed against country level income interquantile ratio data obtained from the World Bank, OECD and World Income Inequality Database. The analysis was stratified by country income (Low, Middle and High) according to the World Bank classification and gender. Multiple regression was used to quantify the association between physical activity and income inequality. Models were adjusted for GDP and percentage of GDP spent on health care for each country and out of pocket health care spent.

**Results:**

Significantly higher levels of inactivity and a wider gap between the percentage of women and men meeting global physical activity guidelines were found in countries with higher income inequality in high and middle income countries irrespective of a country wealth and spend on health care. For example, in higher income countries, for each point increase in the interquantile ratio data, levels of inactivity in women were 3.73% (CI 0.89 6.57) higher, levels of inactivity in men were 2.04% (CI 0.08 4.15) higher and the gap in inactivity levels between women and men was 1.50% larger (CI 0.16 2.83). Similar relationships were found in middle income countries with lower effect sizes. These relationships were, however, not demonstrated in the low-income countries.

**Conclusions:**

Economic inequalities, particularly in high- and middle- income countries might contribute to physical inactivity and might be an important factor to consider and address in order to combat the global inactivity pandemic and to achieve the World Health Organisation target for inactivity reduction.

**Supplementary Information:**

The online version contains supplementary material available at 10.1186/s12966-020-01039-x.

## Background

Physical inactivity is a global pandemic [1] associated with negative physical and mental health outcomes like cardiovascular diseases, type 2 diabetes, obesity, some types of cancer, and depression; and a premature mortality burden estimated at 5.3 million death per annum [2]. Global surveillance data indicate that in 2016 levels of physical inactivity remained high (27.5%) and stable over the previous 10 years, with a worrying eight percentage points gender inequality [3]. At the same time, there is a growing recognition that systemic changes, and not just individual behaviour change, are required to decrease levels of physical inactivity [4–8]. The World Health Organisation (WHO) has set a target for a 10% reduction in physical inactivity by 2025, against which a global action plan was launched in 2018 [9]. As part of this plan, the global physical activity recommendations are updated this year for all age groups [10]. The WHO action plan for physical activity targets multiple factors and seeks synergies within society, societal systems and the environment in innovative ways. However, economic factors are noticeably absent from the WHO action plan and some of the proposed systems thought to determine population levels of physical activity [4]. The impact of the economic context on physical activity has received very little attention, this despite the rise in economic inequality globally and its impact on population health and health inequalities [11].

Therefore, the main aim of this paper is to examine the relationship between within-country income inequality and physical (in)activity and the gender gap in physical activity levels across countries worldwide. This, in order to understand if interventions targeting economic inequality should be investigated within public health campaigns against physical inactivity.

## Methods

### Data sources

Physical activity data were obtained from the World Health Organisation (WHO) estimate pooled from 358 surveys in 168 countries [3]. In this data, insufficient physical activity was defined as adults not meeting the 2010 WHO physical activity guidelines for health [12] —i.e., at least 150 min of moderate-intensity, or 75 min of vigorous-intensity physical activity per week, or any equivalent combination of the two. For this study we used the published age-standardised estimated prevalence [3] in the country as a whole, and for men and women separately, who were not meeting the guidelines for the most recent year available in each country (ranging from 2001 to 2016). The age-standardisation was performed by Guthold et al. to account for the possible influence of difference in age distribution between countries. In addition, we computed the activity gender gap by calculating the difference in estimated compliance to the guidelines between men and women. A higher activity gender gap indicates that within a country more men are meeting the WHO physical activity guidelines than women.

Income inequality, i.e. the difference between those with the highest and lowest incomes in a society, is linked with population health, independent of the income of individuals [11, 13, 14]. Economic inequality was measured as income interquantile ratio (S80/20), which compares the income of the top 20% richest to the poorest 20% within a country [15]. This measure of economic inequality is used by international agencies such as the United Nation, World Bank and OECD. S80/20 data were obtained from the World Bank Development Research Group database [16], the World Income Inequality Database [17] and the OECD Income Distribution Database [18]. For more information about the World Bank methodology see [19]. To ensure that we used only robust data, we cross-referenced data from the WIID OECD and World Bank and excluded data when the estimates were more than 20% apart [20].

Gross Domestic Product (GDP) data were from the World Bank Development research group [16]. Current health care expenditure and out of pocket health care expenditure were also obtained from the World Bank Development research group [16] and cross referenced against the World Health Organisation Global Health Expenditure Database [21].

We used inequality, GDP and health care spent data concurrent to the year of the physical activity estimates for each country or from the closest previous year when not available.

### Analysis

We used multiple regression to investigate the relationship between income inequality and insufficient physical activity levels at country level. We stratified the analysis by World Banks income group [22]. In the models, the dependent variables were the percentage of the whole, male and female population who are inactive and the activity gender gap. We first estimated their association with S80/20 adjusting for each country GDP so that the association reflected the relationship with inequality adjusted for country’s wealth. We then further adjusted the models for country level and out of pocket health care expenditure. We conducted sensitivity analysis by removing 10% of the data in each income group and repeating the analysis. All models were checked for compliance with assumptions necessary for multiple linear regression.

## Results

**Fig. 1:**
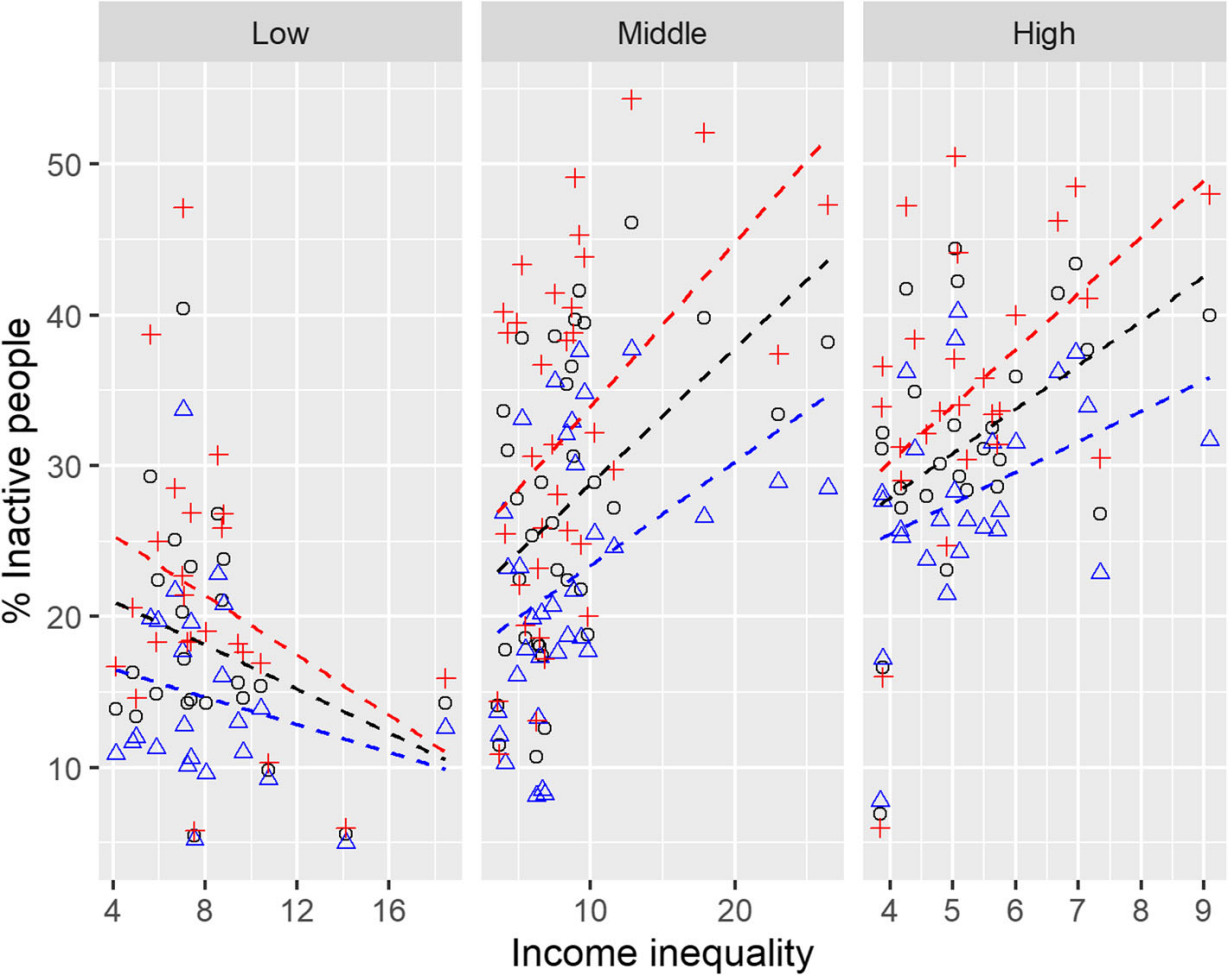
Relationship between insufficient physical activity and income inequality by World bank income group. Whole country population data are in black (o), male data are in blue (Δ) and female in red (+). Raw association are plotted as dashed line with the same colour coding

In total, full data were available for 84 countries (24 from low income, 34 from middle income and 26 from high income countries). Summary statistics are given per income group in Table 1. The relationship between insufficient physical activity and income inequality is depicted graphically in Fig. 1, with raw regression line in black for whole population, blue for male population and red for female population level of insufficient physical activity. An additional plot showing the country names is available in the supplementary materials.

**Table 1:** Summary statistics for insufficient physical activity levels, activity gender gap and income inequality by country income group, given as median and interquartile range

Country Income group	Low	Middle	High
Whole population insufficient physical activity (%)	15.5 (14.3 22.6)	27.5 (18.7 36.3)	31.1 (28.4 37.3)
Male population insufficient physical activity (%)	12.7 (10.8 19.6)	21.2 (17.37 28.8)	27.4 (25.4 31.7)
Female population insufficient physical activity (%)	18.7 (16.9 26.1)	31.8 (23.6 40.4)	33.95 (31.3 40.8)
Activity gender gap (%)	6.30 (3.30 8.30)	9.45 (6.33 15.5)	7.25 (4.38 9.50)
S80/20	7.37 (6.50 8.95)	7.44 (5.60 9.31)	5.06 (4.29 5.74)
Health care expenditure (% of GDP)	6 .00 (5.00 8.00)	6.00 (4.25 7.75)	9.00 (8.00 10.0)
Out of pocket (% health expenditure)	42.0 (31.5 61.0)	32.5 (23.6 40.4)	16.0 (14.0 22.5)

**Fig. 2:**
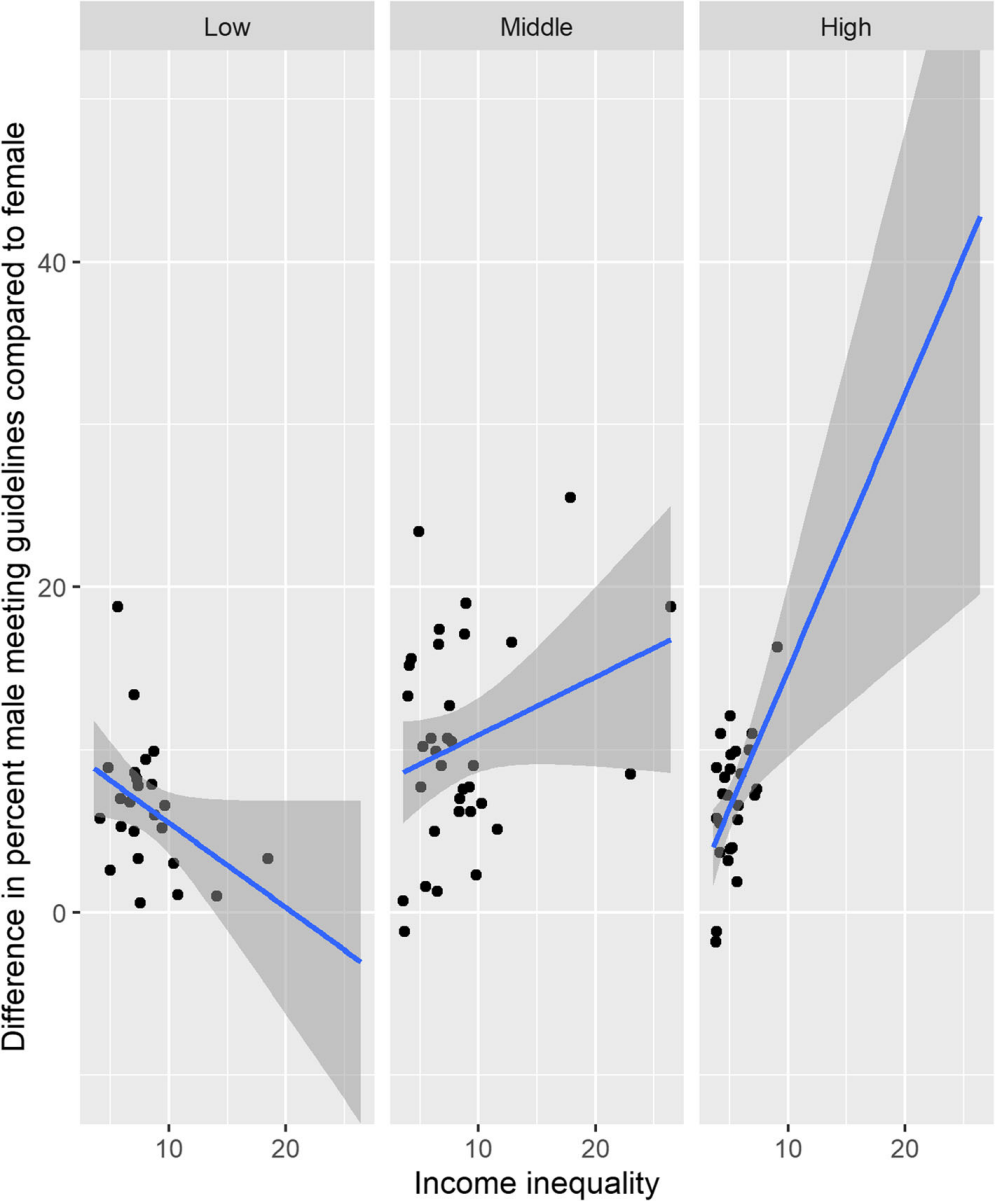
Relationship between gender activity gap and Income inequality per World Bank country income group. Blue lines show the trend with 95% confidence intervals shown as grey ribbons. The income inequality scale are kept identical between plots to facilitate comparision

Figure 1 shows that there is a trend toward higher insufficient physical activity levels in countries with higher income inequalities in middle and high income countries. The gradient of this relationship appears steeper for women than men. This becomes more apparent in Fig. 2 which shows that in high and middle income countries, the gender activity gap is higher in those countries with higher income inequalities. The linear regression confirmed that the relationships in Figs. 1 and 2 were statistically significant at *p* < 0.05 level for both high and middle income countries (Table 2) and independent of country wealth, as measured by GDP. Adjusting for health care expenditure and out of pocket health care expenditure did not change these relationships. Models explained around 20% of the variance in insufficient physical activity for high and middle income countries. The model predicts that if there was no income inequality the residual insufficient physical activity would be 12 to 15% in high and middle income countries and the gender activity gap would disappear.

**Table 2:** Multiple regression unadjusted coefficients with 95% confidence intervals. Significant associations at *p* < 0.05 are highlighted in bold

Country Income Group	High	Mid	Low
Raw association			
Whole population	**2.93 (0.48 5.37)**	**0.90 (0.28 1.52)**	−0.73 (− 1.78 0.32)
Male	**2.04 (0.08 4.15)**	**0.69 (0.12 1.26)**	−0.45 (− 1.35 0.44)
Female	**3.73 (0.89 6.57)**	**1.10 (0.35 1.83)**	−0.99 (−2.24 0.26)
Activity gender gap	**1.70 (0.60 2.80)**	**0.36 (0.09 0.80)**	−0.55 (−1.06 0.02)
Models adjusted for health care expenditure			
Whole population	**3.00 (0.17 5.83)**	**0.85 (0.18 1.51)**	−0.56 (−1.69 0.57)
Male	**2.20 (0.21 4.62)**	**0.55 (0.03 1.13)**	−0.34 (−1.31 0.63)
Female	**3.70 (0.39 7.02)**	**1.11 (0.31 1.92)**	−0.77 (−2.11 0.57)
Activity gender gap	**1.50 (0.16 2.83)**	**0.54 (0.10 0.97)**	−0.43 (− 0.16 1.02)

Repeated analysis with 10% of the data removed by country income level performed as a sensitivity analysis did not change the models significantly (Supplementary material Table S2). The sensitivity analysis showed that the relationships were robust.

## Discussion

Our results show that the levels of insufficient physical activity in high and middle-income countries are higher where there are greater income inequalities, regardless of the country wealth or health care expenditure. In addition, the gender inequality in terms of physical activity levels is also greater in high- and middle- income countries with higher income inequalities. Conversely, these trends are flatter and even inverse in low-income countries. The models estimate that for a point increase in income inequality there is a 3% increase in insufficient physical inactivity for the whole population, 2.2% for men and 3.7% for women in high income countries. A point different in income inequality is roughly the difference between income inequality in France and the United Kingdom. Considering that the difference in insufficient physical activity levels between these countries is around 6%, income inequality could account for half this difference. Similarly compared to the 5% increase in insufficient physical activity observed in high income countries by Guthold et al. [3] over 15 years the estimated effect size of income inequality appears large. The association is about a third weaker in middle income countries.

Our findings extrapolate on a global scale what has already been observed within the USA. Two studies previously examined the association between income inequality and physical (in)activity between states in the USA [23, 24]. Diez-Roux and colleagues found that state-level inequality was associated with higher levels of physical inactivity (i.e. not meeting the physical activity guidelines) in US adults [23]. Similarly, another USA study found that state-level income inequality was associated with a lower likelihood of meeting the physical activity recommendations, but only among women [24].

Many studies have demonstrated that people living in areas with high income inequality are at higher risk for premature mortality and other negative health outcomes, like cardiovascular diseases, depression, obesity and lower self-rated health as demonstrated [25–27]. Considering the physical inactivity is associated with poor health, it is possible that inequality in physical activity plays an important role the relationship between income inequality and poor health.

Several hypotheses exist to explain why income inequality contributes to poorer health outcomes. On the one hand, the social capital hypothesis posits that income inequality intensifies social hierarchies and social inequality and consequently reduces population health through decreased interpersonal trust, social cohesion and social capital [13, 28]. On the other hand, the neomaterialist hypothesis claims that income inequality leads to underinvestment in health services and infrastructures, and education, and thus to poorer health [29, 30].

Income inequality might be an important determinant of insufficient physical activity and poor health that needs to be more clearly understood and taken into account. Negative associations between income inequality and health outcomes are usually found in large areas like countries or states, while findings are less consistent at the level of cities, counties or neighbourhoods [31–34]. This suggests that income inequality could be acting on large systemic scale and at societal level, hinting that high level policy measures are required to address it. For example, the Hass Institute proposed six evidence based policy solutions to reverse inequality: increase minimum wage, expand the earned income tax, build assets for working families, invest in education, make tax more progressive, end residential segregation [35]. Reducing income inequality might be an important lever to increase global physical activity and physical activity equity and prevent raise in inactivity and activity gender gap as countries transition from low income to middle- and high- income economies. Alternatively, it is possible that finer grained analyses are better equipped to account for confounders. However, small geographical scale analysis might not allow to capture macroscopic systemic effects.

Another possible explanation for differences in association between income inequality in high and middle income settings versus low-income countries, may be related to differences in the nature of physical activity and whether it is volitional or utilitarian physical activity. Stalsberg and Pedersen recently showed that the only consistent relationship between self-reported physical activity and socioeconomic status (SES) was for recreational or leisure-time physical activity, and that while persons with low SES did not have resources to direct to leisure time physical activity, they were more actively engaged in physical activity in other domains [36]. This supports the findings by Guthold et al. (2011) of 22 countries in the African region, most of which were low income countries, in which over 79% were meeting WHO global physical activity recommendations [37]. However, the vast majority of physical activity was utilitarian, in the form of occupational (48.6%) or transport-related (46.3%) physical activity, with only 5.3% accounted for by leisure time activities. The gender gap was also greatest for leisure time activity, and any form of vigorous activity. Similarly, Atkinson et al. found marked differences in inactivity levels in low income countries according to occupational structure [38].

In considering why income inequality has a differential effect on levels of physical activity in high- and middle-income countries, compared to low income countries, we can think of the broader upstream factors, such as infrastructure and access to facilities and resources, or social determinants such as safety from crime. In a recent narrative review, Adkins et al. suggested that in low-income, socio-economically disadvantaged communities, the associations between the built environment and physical activity are weaker and often inverse [39]. This is supported by a number of studies. For example, Da Silva (2014) demonstrated in a study of over 100,000 Brazilian adolescents, from more than 2800 schools that physical activity in adolescents was inversely associated with income inequality of the city in which they lived [40]. Da Silva suggests that one way in which to address this social gradient, or “level the playing fields” in cities would be to develop the infrastructure to promote physical activity for the entire population, through constructing “free-access public areas” such as parks, sports fields, recreation facilities, and greenways.

In terms of other attributes of the built environment, residential density in high -income countries and the Global North has been associated with increased walking for leisure [41]. Conversely, densification in low-income country settings reflects overcrowding and has little or no association, and may, in fact, be negatively associated with walking for leisure [42]. The results of the current study suggest that it is important to consider both the volitional and utilitarian nature of physical activity, infrastructure, programmes, densification and the quality of what comprises the urban space, against the needs of the inhabitants and their perceptions [43].

To date the promotion of physical activity has mainly focused on individual behaviour change, community-based interventions and sport promotion [8] with only limited impact on global trends in inactivity [3]. Some might argue that the focus on sport might be counter-productive as it possibly re-inforces social hierarchy and hence the potential effect of inequality on inactivity above [44]. It is also possible to raise the question of whether physical inactivity might be a symptom of inequality (a consequence), rather than mainly a behavioural issue exacerbated in high income countries. Proximal determinants targeted by most behavioural interventions only explain a small proportion of the variance in population physical activity [45, 46]. Conversely, it appears that income inequality could explain around 20% of the variance suggesting that it could be a much more powerful lever. Recent report show that we are not on course to meet the WHO target for a 10% reduction in physical inactivity [3]. Our model estimates that 10% lower physical inactivity is associated with an income inequality lowered by a factor of 3 in high income countries. For example, this would be equivalent to bring levels of inequality in the USA to the levels observed in Norway which Norway as a country show it is feasible. Considering the $53.8 billion economic burden and $13.7 billion productivity loss associated with physical inactivity, which are probably underestimated, reducing income inequality might make economic as well as public health sense [47].

### Strength and limitations

The main strength of this study is that we used data openly available from international agencies such as the WHO, UN and World Bank and cross-referenced them. The main limitations are with the quality of the data. Measures of income inequality are also notoriously imprecise [15]. In addition, it is recognised that wealth inequality might be a stronger driver of outcome inequality than income inequality. Nonetheless, the relationships observed are quite apparent without the need of complex analysis which give some support to their robustness. Finally, this is only a cross-sectional analysis therefore it is not possible to offer definitive answer about whether these are causal relationships or simply concurrent phenomena.

## Conclusion

Physical inactivity levels and gender gap in activity levels are strongly related to within country income inequality in high and middle income countries. Economic inequalities might contribute to the global pandemic of physical inactivity and might be an important factor to consider and address in order to achieve the World Health Organisation target for inactivity reduction and combat the pandemic and its associated burden of disease and mortality.

## Supplementary Information

**Additional file 1: Fig. S1:** Relationship in low income countries between within country income inequality and percentage of people inactive within a country. Country code are presented and can be cross-referenced with real names in Table S1. The linear raw association is presented with 95% CI ribbon. **Fig. S2:** Relationship in middle income countries between within country income inequality and percentage of people inactive within a country. Country code are presented and can be cross-referenced with real names in Table S1. The linear raw association is presented with 95% CI ribbon. **Fig. 3:** Relationship in high income countries between within country income inequality and percentage of people inactive within a country. Country code are presented and can be cross-referenced with real names in Table S1. The linear raw association is presented with 95% CI ribbon. **Table S1:** Country code and name reference table. **Table S2:** Results of sensitivity analysis.

## Data Availability

All data used in this manuscript are freely available, the references are given in the main text.
