# Supplementary material for: Inequality in physical activity, global trends by income inequality and gender in adults
Source: Int J Behav Nutr Phys Act. 2020 Nov 26;17:142. doi: 10.1186/s12966-020-01039-x (PMC7690175; doi:10.1186/s12966-020-01039-x)
Supplement: Supplementary file 1 — Additional file 1: Fig. S1: Relationship in low income countries between within country income inequality and percentage of people inactive within a country. Country code are presented and can be cross-referenced with real names in Table S1. The linear raw association is presented with 95% CI ribbon. Fig. S2: Relationship in middle income countries between within country income inequality and percentage of people inactive within a country. Country code are presented and can be cross-referenced with real names in Table S1. The linear raw association is presented with 95% CI ribbon. Fig. 3: Relationship in high income countries between within country income inequality and percentage of people inactive within a country. Country code are presented and can be cross-referenced with real names in Table S1. The linear raw association is presented with 95% CI ribbon. Table S1: Country code and name reference table. Table S2: Results of sensitivity analysis. [file 12966_2020_1039_MOESM1_ESM.docx]

Supplementary material

**Title: Inequality in physical activity. Global trends by income inequality and gender**


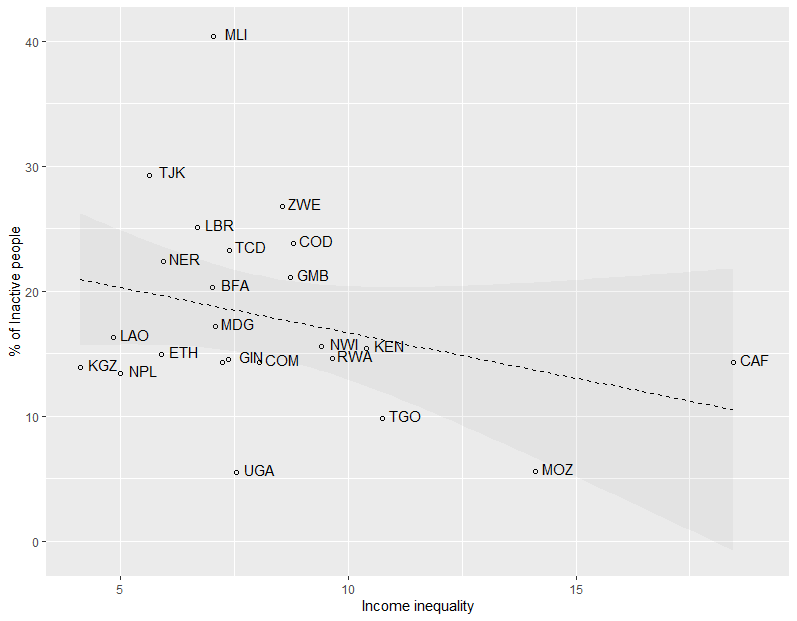
This supplementary material contains copies of the plots in Figure 1, split by income level and including country’s label. Labels can be cross-referenced to country’s name in Table S1 below.

Figure S1: Relationship in low income countries between within country income inequality and percentage of people inactive within a country. Country code are presented and can be cross-referenced with real names in Table S1. The linear raw association is presented with 95% CI ribbon.


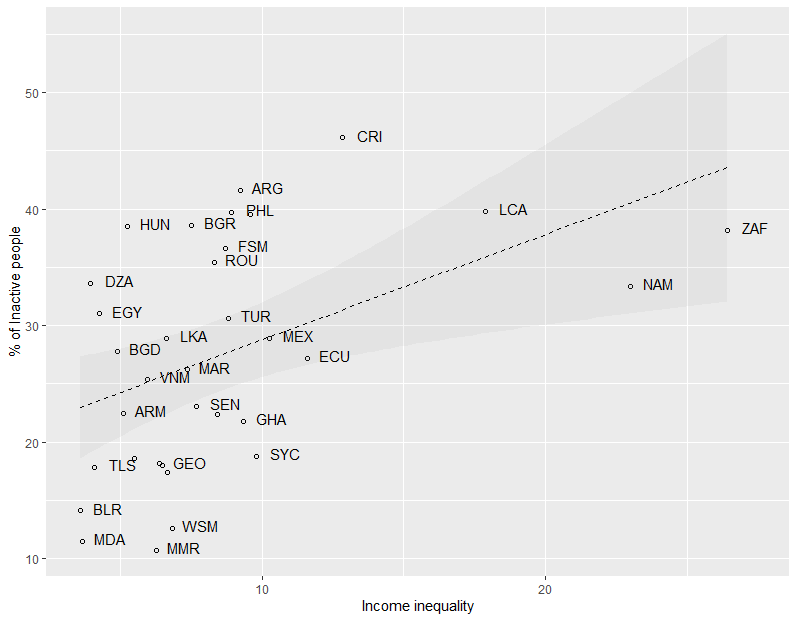


Figure S2: Relationship in middle income countries between within country income inequality and percentage of people inactive within a country. Country code are presented and can be cross-referenced with real names in Table S1. The linear raw association is presented with 95% CI ribbon.


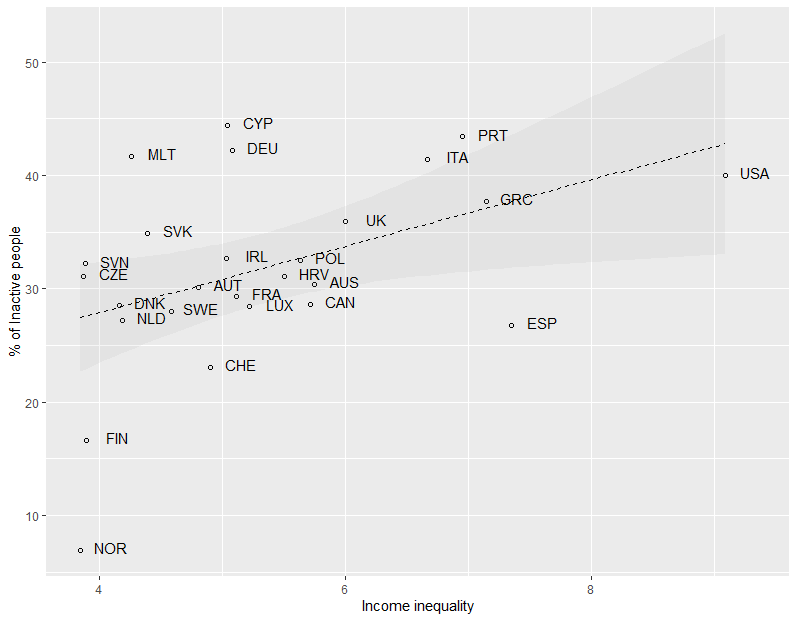


Figure 3: Relationship in high income countries between within country income inequality and percentage of people inactive within a country. Country code are presented and can be cross-referenced with real names in Table S1. The linear raw association is presented with 95% CI ribbon.

**Table S1: Country code and name reference table.**

| Country | Country_Code |
| --- | --- |
| Algeria | DZA |
| Argentina | ARG |
| Armenia | ARM |
| Australia | AUS |
| Austria | AUT |
| Bangladesh | BGD |
| Belarus | BLR |
| Bulgaria | BGR |
| Burkina Faso | BFA |
| Canada | CAN |
| Central African Republic | CAF |
| Chad | TCD |
| Comoros | COM |
| Costa Rica | CRI |
| Croatia | HRV |
| Cyprus | CYP |
| Czech Republic | CZE |
| Democratic Republic of the Congo | COD |
| Denmark | DNK |
| Ecuador | ECU |
| Egypt | EGY |
| Ethiopia | ETH |
| Finland | FIN |
| France | FRA |
| Gambia | GMB |
| Georgia | GEO |
| Germany | DEU |
| Ghana | GHA |
| Greece | GRC |
| Guinea | GIN |
| Hungary | HUN |
| Ireland | IRL |
| Italy | ITA |
| Kenya | KEN |
| Kyrgyzstan | KGZ |
| Lao | LAO |
| Liberia | LBR |
| Luxembourg | LUX |
| Madagascar | MDG |
| Malawi | NWI |
| Mali | MLI |
| Malta | MLT |
| Mexico | MEX |
| Micronesia | FSM |
| Mongolia | MNG |
| Morocco | MAR |
| Mozambique | MOZ |
| Myanmar | MMR |
| Namibia | NAM |
| Nepal | NPL |
| Netherlands | NLD |
| Niger | NER |
| Norway | NOR |
| Philippines | PHL |
| Poland | POL |
| Portugal | PRT |
| Republic of Moldova | MDA |
| Romania | ROU |
| Rwanda | RWA |
| Saint Lucia | LCA |
| Samoa | WSM |
| Senegal | SEN |
| Serbia | SRB |
| Seychelles | SYC |
| Sierra Leone | SLE |
| Slovakia | SVK |
| Slovenia | SVN |
| Solomon Islands | SLB |
| South Africa | ZAF |
| Spain | ESP |
| Sri Lanka | LKA |
| Sweden | SWE |
| Switzerland | CHE |
| Tajikistan | TJK |
| Timor-Leste | TLS |
| Togo | TGO |
| Tonga | TONFt |
| Turkey | TUR |
| Uganda | UGA |
| United Kingdom of Great Britain and Northern Ireland | UK |
| United States of America | USA |
| Uruguay | URY |
| Viet Nam | VNM |
| Zimbabwe | ZWE |

Sensitivity analysis

**Table S2: Results of sensitivity analysis**

| **Country Income Group** | **High** | **Mid** | **Low** |
| --- | --- | --- | --- |
| Raw association | | | |
| Whole population | **2.70 (0.15 5.32)** | **0.85 (0.20 1.49)** | -0.42 (-1.33 0.49) |
| Male | **1.82 (0.05 4.14)** | **0.62 (0.04 1.21)** | -0.21 (-0.96 0.53) |
| Female | **3.47 (0.46 6.49)** | **1.04 (0.29 1.81)** | -0.62 (-1.75 0.51) |
| Activity gender gap | **1.65 (0.52 2.78)** | **0.37 (0.07 0.81)** | -0.39 (-1.01 0.23) |
| Models adjusted for health care expenditure | | | |
| Whole population | **2.80 (0.27 5.80)** | **0.80 (0.12 1.48)** | -0.55 (-1.51 0.41) |
| Male | **2.01 (0.06 4.67)** | **0.50 (0.03 1.10)** | -0.36 (-1.13 0.41) |
| Female | **3.41 (0.08 6.90)** | **1.07 (0.25 1.89)** | -0.74 (-1.97 0.48) |
| Activity gender gap | **1.39 (0.02 2.77)** | **0.53 (0.09 0.98)** | -0.36 (-1.12 1.04) |
